# Rational choice and situational convergence: an examination of adolescent cyber victimization from an integrated theoretical perspective

**DOI:** 10.3389/fpsyg.2026.1837866

**Published:** 2026-07-28

**Authors:** Da Dong, Xinyi Lu, Liping Yang, Luyue Zhou

**Affiliations:** 1 Department of Psychology, Shaoxing University, Shaoxing, China; 2 Center for Brain, Mind and Education, Shaoxing University, Shaoxing, China; 3 School of Educational Science, Hunan Normal University, Changsha, China

**Keywords:** adolescents, cyber criminology, cyber victimization, lifestyle theory, rational choice theory, routine activity theory

## Abstract

This paper examines the issue of adolescent cyber victimization through the complementary lenses of routine activity theory (RAT) and rational choice theory (RCT), which together form an integrated framework. RAT posits that the occurrence of crime requires the convergence in time and space of three elements: a motivated offender, a suitable target, and the absence of a capable guardian. RCT supplements this by emphasizing that offenders are rational actors who select targets and opportunities based on a cost–benefit calculation. In the cyber environment, adolescents, due to the online lifestyle shaped by characteristics of their developmental stage, often become suitable targets perceived by offenders as offering high reward and low risk, while guardianship in their online activity environments is frequently lacking. Based on this dual theoretical framework, this paper systematically analyzes the formation mechanisms of adolescent cyber victimization and proposes a multi-dimensional prevention system. This system encompasses three dimensions: raising the cost and threshold of offending, enhancing multi-party guardianship, and disrupting situational convergence. It aims to effectively mitigate the risks of adolescent cyber victimization and safeguard their information security, as well as personal and property safety.

## Introduction

The rapid diffusion of the Internet has increased adolescents' online engagement and, concomitantly, incidents of cyber victimization, making this a pressing social and theoretical problem. Empirical and policy literatures advance technical protections, psychological interventions, and legal reforms; they also frequently invoke routine activity theory (RAT) and rational choice theory (RCT) to account for situational conditions and offender decision-making in cybercrime (Clarke, 1997; Cohen and Felson, 1979). Yet much of this work is descriptive or prescriptive; there is no widely adopted theoretical framework that integrates the developmental profile of adolescents with the situational and offender-centered mechanisms that generate online victimization. This lacuna undermines efforts to design coherent, multi-level prevention strategies tailored to youth.

RAT explains victimization in terms of the spatiotemporal convergence of three elements: a motivated offender, a suitable target, and the absence of a capable guardian (Cohen and Felson, 1979; Felson and Cohen, 1980). More fundamentally, RAT points to lifestyle—habitual patterns of activity and association—as the proximate determinant of exposure to risky situations, the kinds of contacts one encounters, and the degree of guardianship one receives (Leukfeldt and Yar, 2016). The three RAT elements do not operate independently; lifestyle shapes exposure and self-protection, and these in turn affect perceived target suitability. In online settings, a single behavioral tendency (e.g., extensive self-disclosure) can both increase visibility to offenders and weaken self-guardianship, thereby converting latent opportunities into actual victimization. Empirical work supports RAT's applicability to cybercrime broadly and to cyber-interpersonal violence in particular: motivated offenders, suitable digital targets, and deficient guardianship jointly constitute the necessary conditions for many forms of online victimization (Choi and Lee, 2017; Leukfeldt and Yar, 2016).

RCT supplements this situational account by modeling offender agency. It treats offenders as decision-makers who weigh expected costs and benefits and hence select opportunities with favorable payoff–risk profiles (Clarke, 1997). In cyberspace, features such as anonymity, low barriers to access, and weak monitoring lower perceived detection risk and thus alter the offender's calculus (Whitty, 2019). Bounded rationality—the recognition that real agents simplify decisions under cognitive limits or time pressure—explains why offenders adapt tactics to exploit predictable victim behaviors and technological affordances (O'Grady, 2011; Whitty, 2019). Empirical studies of property offenders and cyber-fraudsters corroborate this picture: offenders deliberate over target value, visibility, accessibility, and escape routes, adjusting methods as contexts change (Clarke, 1997).

Applied in isolation, RAT identifies the risk factors and opportunity conditions of victimization but cannot explain why offenders deliberately select particular victims (Aizenkot, 2022; Holt and Bossler, 2008). Applying RCT alone fails to clarify how risky contexts converge spatiotemporally in online environments. The RAT–RCT integrated framework developed in this study may fill this research gap by combining situational opportunity factors with offenders' decision-making processes, enhancing explanatory comprehensiveness. Conceptually, RAT and RCT address complementary aspects of cyber victimization (see Table 1): RAT specifies the structural and behavioral opportunity conditions that make victimization possible; RCT explains how offenders perceive and exploit those conditions. This dual perspective is especially useful for understanding adolescent vulnerability. Adolescence involves neurocognitive and psychosocial transformations—heightened sensation seeking, intensified peer influence, exploratory identity work, and still-maturing inhibitory control—that shape online lifestyles and risk profiles (Balakrishnan et al., 2025; Higgins et al., 2008; Jiang et al., 2025; Zhang et al., 2022). These traits promote behaviors—excessive disclosure, rapid trust in strangers, engagement with high-risk platforms—that increase exposure to motivated offenders, enhance perceived target attractiveness, and commonly occur amid inadequate parental, educational, or technological guardianship. Offenders, operating under bounded rationality, can detect and exploit these predictable patterns by designing low-effort, high-reward schemes targeted at adolescents (Schittenhelm et al., 2025; Whitty, 2019).

**Table 1:** Comparison of implications between RAT and RCT dimensions.

Dimensions	RAT	RCT
Core logic	Crime arises from the spatiotemporal convergence of three elements: motivated offender, suitable target, and absence of a capable guardian	Offenders function as rational decision-makers who select targets and opportunities based on cost–benefit analysis, bounded by limited rationality
Key constructs	•Motivated offender •Suitable target •Absent guardianship	•Perceived costs •Expected benefits •Bounded rationality
Analytical focus	Explains why opportunities for victimization exist (lifestyle, exposure, guardianship)	Explains why offenders choose specific victims/timing (decision-making, target selection)
Limitation alone	Identifies risk opportunities but cannot explain why offenders deliberately target particular victims	Explains offender decision-making but does not clarify how risky situations converge spatiotemporally online
Prevention logic	Disrupt convergence of the three elements; reduce target suitability; strengthen guardianship; limit exposure	Alter offenders' cost–benefit calculus; increase offending costs; reduce expected rewards
Core prevention measures	•Reduce target suitability •Enhance guardianship •Block situational convergence	•Raise offending costs •Lower illicit gains •Deter decision-making
Prevention priority	Reduce vulnerability from external contexts	Block crime through internal decision shifts
Complementarity	Provides the situational foundation	Supplies the decision mechanism

**Figure 1:**
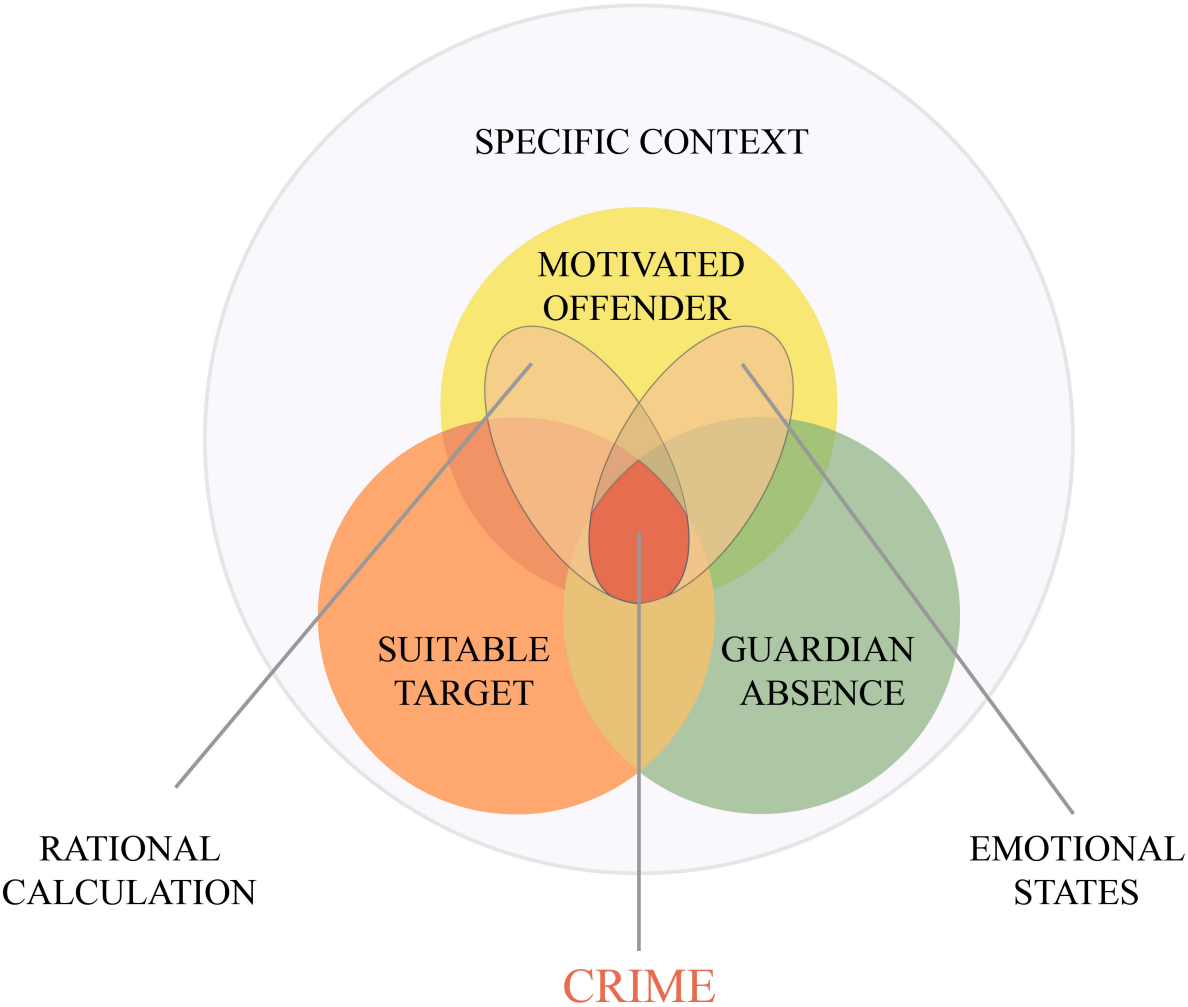
A dual-theory framework for analyzing cyber victimization (based on Clarke, 1997; Cohen and Felson, 1979).

Despite extensions of RAT into digital domains, research that systematically links adolescent cognitive-behavioral development to susceptibility within an integrated RAT–RCT framework remains sparse and fragmented; studies often focus on single victimization types rather than on the shared mechanisms across forms of cyber harm (Griffith et al., 2023). To address this gap, the present paper offers an analytical framework that combines RAT's situational lens with RCT's account of offender deliberation. RAT identifies the environmental and behavioral preconditions of victimization; RCT explicates the offender's selection and exploitation of those preconditions. Together, they clarify how developmental traits, online lifestyles, and guardianship deficits interact with offender decision processes to produce diverse instances of adolescent cyber victimization (see Figure 1). This integrated framework can explain common generative processes for most typical victimization as well as their respective type-specific mechanisms.

The outermost “specific context” circle denotes the socio-technical and temporal environment—platform architectures, algorithms, community norms, legal regimes, and usage settings—that shape how opportunities for harm arise and persist. The yellow “motivated offender” circle represents actors with incentives to offend whose actions reflect both instrumental cost–benefit reasoning and influences associated with bounded rationality, such as limited information and transient emotional states. The orange “suitable target” circle captures adolescents' developmental and behavioral features—excessive disclosure, peer-oriented risk-taking, impulsivity, and limited digital literacy—that increase their perceived value and accessibility to offenders. The green “guardian absence” circle signals lapses or fragmentation in family, school, peer, and platform protections, including weak monitoring, poor reporting pathways, and a lack of cross-sector coordination. Where these colored domains overlap, the small red core marks the high-probability zone for cyber victimization: crime occurs when offender motivation, target suitability, and guardianship gaps coalesce within a given specific context.

This integrated framework has some implications for policymakers. Effective prevention must be multi-level and stakeholder-inclusive, operating through three complementary levers: raising the cost and threshold of offending, enhancing multi-party guardianship, and disrupting situational convergence. By linking developmental psychology, situational opportunity structures, and offender reasoning, the RAT–RCT synthesis aims to guide targeted empirical research and principled, theory-driven interventions against adolescent cyber victimization.

## A dual-theory framework for cyber victimization

Crime does not follow automatically from contact between an offender and a potential victim; many adolescents encounter online threats without becoming victims. Whether an encounter yields harm depends on the interaction of situational opportunity, offender choice, and victim vulnerability. A complete account of cybercrime, therefore, must adopt a two-sided framework that treats opportunity structures and offender agency together with the developmental and behavioral features of adolescents.

### Routine activity and criminal opportunities

Cyber offenders pursue motives such as financial gain, revenge, or distorted psychological gratifications and combine technical competence with psychological adaptability to exploit digital environments. Internet anonymity lowers psychological barriers to offending and enables shifting modus operandi that track social trends (Whitty, 2019). Adolescent habits—visiting high-risk sites, credulously accepting online information, and displaying strong peer conformity—both enlarge contact opportunities and can stimulate offender motivation. For instance, adolescents who flaunt virtual wealth or display emotional volatility may attract fraudsters or manipulators.

RAT's key notion of a “suitable target” emphasizes features that make exploitation likely. For adolescents, these features tie to developmental traits: poor personal information management often reflects a heightened need for self-expression and social validation during a period of sensation seeking and peer conformity, producing excessive disclosure that eases data harvesting (Higgins et al., 2008; Zhang et al., 2022). Limited self-protection awareness and capacity trace to ongoing prefrontal development; adolescents may not reliably detect phishing or manipulation, which reduces vigilance. Body dissatisfaction further undermines self-guardianship and monitoring, increasing vulnerability (Malinowska-Cieślik et al., 2022). Risky behaviors—trusting strangers, clicking unknown links, engaging in unprotected intimate exchanges—may reflect sensation seeking, peer conformity, and insufficient self-regulation, thereby heightening both exposure and exploitability (Balakrishnan et al., 2025; Jiang et al., 2025; Zhang et al., 2022).

Capable guardianship—informal (family, peers, teachers) or formal (law enforcement, platform moderators)—is often weak online. Technical shortcomings (weak passwords, poor authentication, inadequate encryption, and sparse auditing) lower the effort required for crime and delay detection (Leukfeldt and Yar, 2016). Social guardianship is impaired when parents and teachers lack digital literacy or awareness of adolescents' online lives, limiting effective supervision. Platform operators likewise may fail in moderation, behavioral monitoring, and rapid response (DeLiema et al., 2025). These technical and social deficits increase the likelihood that suitable adolescent targets and absent guardianship will co-occur—the core RAT conditions for cyber victimization. However, these three factors are framed from the victim's perspective; we lack the complementary dimension that examines the crime process from the offender's point of view.

### Rational choices and criminal opportunities

As shown in Figure 1, routine activity theory attributes cybercrime to the spatiotemporal convergence of a motivated offender, a suitable target, and absent guardianship, while rational choice theory complements this by explaining how, within digitally mediated routines and platform affordances, offenders identify and exploit opportunities through cost–benefit calculations shaped by bounded rationality and emotional influences. RCT models offenders as decision-makers who scan contexts for opportunities and weigh costs against benefits. Digital affordances—anonymity, easy access, and weak guardianship—lower perceived detection risk and psychological restraint, creating niches where crime is attractive (Whitty, 2019). Offenders employ bounded rationality to simplify choices under cognitive limits or time pressure and thus target contexts (social media, online games, chatrooms) where adolescents routinely disclose exploitable information and where automated tools can efficiently harvest targets (Whitty, 2019).

RCT complements RAT by explaining how offenders exploit routine-generated opportunities: tactics are adapted to platform affordances and monitoring gaps—for example, phishing campaigns on emerging apps with weak moderation. Contextual variables (time of day, platform norms) shape offender calculations and point to targeted environmental interventions—target hardening (stronger passwords), access controls (parental filters), and temporal restrictions—that raise effort and perceived risk (Clarke, 1997).

Emotions modify the rational calculus. Bounded rationality concedes that emotional arousal can distort cost–benefit reasoning, increasing focus on immediate rewards and diminishing attention to long-term consequences (Elster, 1986; Kaufman, 1999). Anger, excitement, or the illicit thrill of transgression can precipitate impulsive acts where perceived costs shrink (Simpson, 2000). Anonymity in online settings can amplify this detachment, eroding offenders' recognition of victims as persons rather than mere targets (Dong and Chen, 2025). Adolescents' emotional vulnerabilities, partly grounded in shared social-cognitive and mirroring mechanisms (Dong et al., 2025), both increase their attractiveness as targets and provide exploitable leverage for offenders (Fabris et al., 2022; Jiang et al., 2025).

Rational calculation in cybercrime thus involves estimating success probability, punishment severity, and expected benefit. Offenders favor targets marked by naivety, excessive trust, weak cybersecurity, or high visibility—traits common among adolescents—because these reduce effort and perceived risk (Whitty, 2019). Guardianship deficits further lower perceived costs and encourage selection of such targets (Leukfeldt and Yar, 2016). Bounded rationality and time pressure also amplify susceptibility in loss-framed scenarios, which attackers exploit (Lyu et al., 2025).

For prevention, analyzing offender calculation suggests measures that increase perceived costs and effort (surveillance, sanctions, publicized penalties, technical barriers) and reduce rewards. Because offenders adapt, prevention must be dynamic: monitor criminal strategies, update defenses, run awareness campaigns, and strengthen guardianship across family, schools, and platforms (Clarke, 1997).

### Interactive mechanisms in adolescent cyber victimization

Importantly, adolescent victimization does not arise from any single condition in isolation but from the accumulation and spatiotemporal overlap of these elements—the red intersection in Figure 1—where developmental vulnerabilities, risky online routines, and deficits in guardianship converge and become exploitable by offenders acting on favorable cost–benefit calculations. Applied to adolescent cyberbullying, RAT and RCT jointly illuminate how situational opportunity and offender choice interact. RAT specifies the necessary convergence—motivated offender, suitable target, and absent guardianship—while RCT explicates how perpetrators select targets and timing by assessing costs and benefits (Clarke, 1997). Together, they explain both why opportunities arise and why certain opportunities are seized.

Recent empirical work across diverse national contexts converges on the view that adolescent online victimization shares common underlying mechanisms while remaining sensitive to local conditions. A cross-national meta-analysis of longitudinal studies confirms that cyber-victimization is reliably associated with subsequent mental health symptoms—particularly depression and anxiety—among children and adolescents, with cultural differences not moderating the effect (Lee et al., 2026). Evidence from East Asian samples supports the framework's situational logic: among Korean youth, online risky behaviors and weak cybersecurity management—markers of heightened target suitability and deficient guardianship—predict both cyberbullying and traditional bullying victimization (Choi et al., 2019). A cross-sectional study of Chinese adolescents during the COVID-19 pandemic further shows that contextual stressors such as epidemic perception shape cyberbullying involvement, underscoring how situational conditions modulate risk (Feng et al., 2024). Collectively, empirical findings from these non-Western jurisdictions furnish suggestive empirical evidence for the functional logic of the integrated RAT–RCT framework across diverse geographic contexts, partially substantiating the cross-cultural explanatory capacity of the integrated theory; meanwhile, such results underline that the prevention and mitigation of online victimization requires a balanced combination of universal preventive principles and contextual local realities to develop differentiated prevention frameworks. Perpetrators use publicly available cues to evaluate a target's resistance and the likelihood of intervention, then choose platforms or time windows with weaker guardianship to maximize gain and minimize risk (Whitty, 2019). This is a form of bounded rationality: perpetrators do not optimize over complete information but seek satisficing options—that is, options that are “good enough” rather than optimal—based on accessible evidence, such as a target's susceptibility or perceived laxity of moderation (O'Grady, 2011). Concurrently, adolescents' cognitive limits and emotional states can erode self-guardianship, producing the “absent guardianship” RAT identifies (Jiang et al., 2025). Anonymity further lowers the perceived costs of bullying, while adolescents' persistent activity traces increase the probability of encounter. Empirical studies show that perpetrators exploit periods of emotional vulnerability—when adolescents' psychological defenses are reduced—to initiate attacks (Griffith et al., 2023).

Rational choice theory critically supplements this baseline by elucidating why exposure to high-risk environments does not invariably culminate in victimization: offenders must actively capitalize on these conditions through strategic cost–benefit evaluations. Consequently, even adolescents with risky lifestyles may evade harm if perpetrators perceive the operational effort as outweighing the illicit reward—a contingent, high-risk but unvictimized state that escapes the explanatory scope of routine activity theory alone. The integrated RAT–RCT framework thus unfolds as a dynamic, transactional mechanism, wherein adolescent behavioral routines generate latent opportunity spaces, and offender decision-making selectively actualizes and amplifies those opportunities. Effective prevention must therefore target both sides of this coupling—altering adolescents' exposure and self-protection capacities and disrupting the offender's evaluative logic—to break the cycle of cyberbullying.

This integrated framework can not only interpret single types of online victimization but also systematically analyze multiple forms of adolescent online victimization, including cyberbullying, online fraud, online sexual victimization, and personal privacy leakage. Most forms of adolescent online victimization share core mechanisms: the spatiotemporal coupling of motivated offenders, suitable targets, and inadequate guardianship, together with offenders' cost–benefit evaluation under bounded rationality. Meanwhile, differences exist across victimization types in terms of offending motives, target characteristics, and manifestations of guardianship deficiencies. Specifically, cyberbullying perpetrators are mostly driven by emotional catharsis, peer recognition, or retaliatory motives, targeting socially vulnerable and emotionally expressive adolescents; guardianship deficiency is reflected in delayed platform moderation and peer bystander behavior, with perpetrators focusing more on social risks than legal sanctions (Choi and Lee, 2017). Online fraud perpetrators are motivated primarily by economic gain, targeting adolescents with relatively weak risk discrimination abilities; inadequate guardianship manifests as insufficient payment security protection and deficient family risk education, and perpetrators' rational judgments center on profit scale and behavioral concealment (Balakrishnan et al., 2025; Schittenhelm et al., 2025; Whitty, 2019). Perpetrators of online sexual harassment and privacy violations are mainly motivated by psychological manipulation and information monetization, closely associated with excessive disclosure of private information by adolescents. Guardianship deficiency stems from insufficient privacy protection education and platform security vulnerabilities, and perpetrators mostly reduce behavioral risks through information asymmetry (Aizenkot, 2022; Leukfeldt and Yar, 2016).

Notably, existing research (Burden, 2025; Deryol and Wilcox, 2020; Kerstens and Jansen, 2016; Weulen Kranenbarg et al., 2019; Xu, 2025) on the victim–perpetrator overlap has frequently been confined to descriptive or correlational analyses; this integrated framework fills this gap by offering a cohesive theoretical perspective that systematically accounts for the explicit generative mechanisms producing these overlapping roles among adolescents. Such transformative cases are prevalent in real life. A systematic review of role dynamics in bullying and cyberbullying concludes that victim and aggressor roles frequently overlap and can be exchanged, with some victims retaliating against their aggressors online and thereby becoming cyberaggressors themselves (Estévez et al., 2020). In addition, there are numerous typical cases in reality where minors steal their parents' accounts to make large purchases of virtual goods, such as online live streaming rewards, game top-ups, and software payments, which can more intuitively reflect the overlapping identity of victims and perpetrators. In the early stage, Internet platforms merely regarded adolescents as pure victims and only adopted one-time real-name authentication, failing to curb behaviors including account theft, unauthorized password recording, and unauthorized use of others' payment credentials. Viewed through this study's integrated framework and research on cyber financial crime, these minors occupy an ambiguous position that blurs the victim–perpetrator boundary: their unauthorized transactions impose financial costs on others even though the minors are not offenders in any criminal sense (Kerstens and Jansen, 2016). Unauthorized consumption relying on the concealment of online identities weakens minors' awareness of property risks. Over time, such experiences may distort minors' perception of property and online risk and, absent guidance, contribute to further risky online behavior.

From a RAT perspective, many victimized adolescents remain exposed to high-risk online environments over long periods, accompanied by extensive personal information disclosure, insufficient external supervision, and frequent contact with potential offenders (Aizenkot, 2022; Holt and Bossler, 2008). Such circumstances give rise to retaliatory and imitative behaviors. In the absence of guardianship, victims are prone to transitioning into perpetrators, which in turn facilitates the recoupling of the three core elements (Averdijk et al., 2016). From the perspective of RCT, such role transformation stems from cost–benefit analysis under bounded rationality: adolescents perceive extremely low costs of conducting retaliatory acts via the anonymity of cyberspace, while gaining rewards including emotional catharsis, peer recognition, and reconstruction of self-worth (Elster, 1986; Kaufman, 1999). Meanwhile, emotional disturbances induced by prior victimization experiences accelerate this role shift (Simpson, 2000).

In addition, there is substantial internal heterogeneity among adolescents, so differences in age, gender, and situational context need to be incorporated into the theoretical framework. In terms of age, early adolescents aged 12–14 have lower self-control and digital literacy and therefore higher target suitability. Adolescents aged 15–18 are more susceptible to peer influence, more likely to be exposed to risky situations, and experience role transition (Higgins et al., 2008). In terms of gender, female adolescents are more vulnerable to online sexual harassment, privacy leakage, and emotional manipulation, while male adolescents are more likely to encounter gaming fraud and engage in cyberbullying perpetration, which is associated with gendered patterns of Internet use (Choi and Lee, 2017; Xu, 2025). In terms of situational context, the lack of guardianship is more prominent in highly interactive scenarios such as social media platforms, online games, and live streaming. In anonymous online environments, offenders exhibit lower risk perception, leading to a significant increase in the likelihood of victimization (Leukfeldt and Yar, 2016; Whitty, 2019).

## A multi-dimensional prevention system

Building on the integrated RAT–RCT analysis, prevention should be framed as a coordinated, multi-level architecture that simultaneously reshapes offenders' cost–benefit calculations and alters the situational conditions that render adolescents suitable targets. Concretely, this architecture comprises three complementary levers—raising the perceived costs and thresholds of offending, enhancing multi-party guardianship, and disrupting situational convergence—and these levers must operate in concert because changing offender rationale without modifying exposure, or vice versa, leaves critical victimization pathways intact. Practical measures include enforcing age limits and synchronizing platform flags with parental and school review to preserve guardianship continuity and raise perceived offender risk (Leukfeldt and Yar, 2016; Sobol et al., 2025); strengthening traceability, platform liability, and anti-monetization measures to increase expected costs; and deploying big-data early-warning systems that analyze routine-activity traces to detect emerging high-risk interactions and trigger timely interventions (alerts to parents, platform moderation, or school outreach) while carefully managing privacy, false positives, and transparent thresholds. Empirical and theoretical work suggests the value of this integrated approach: disrupting the early stages of exploitation can reduce successful attacks (Griffith et al., 2023), and interventions that alter perceived costs and benefits can reshape offender choice (Clarke, 1997).

### Raising the cost and threshold of offending

RCT implies that interventions that change perceived costs and benefits will alter offenders' choices. Conceptually, then, weakening criminal motivation is a matter of changing the instrumental calculus that makes offending attractive. Policy levers include (i) increasing perceived detection and sanction probabilities—by strengthening traceability, reinforcing platform legal liability, and improving digital forensics—and (ii) reducing expected gains—by disrupting illicit monetization channels (e.g., fund interception, encrypted-data protections) (Whitty, 2019). Requiring stricter identity verification on platforms and publicizing successful prosecutions are practical instantiations: they raise the expected cost of offending and thus shift the offender's expected utility toward non-offending. These measures operate instrumentally to reshape rational appraisal; empirical work on cyber fraud supports their capacity to disrupt offenders' risk–reward assessments (Griffith et al., 2023).

RAT locates prevention in the qualities of potential targets and their routines (Cohen and Felson, 1979). Reducing adolescents' suitability, therefore, requires interventions that lower attractiveness, visibility, and accessibility. Educational programs that improve digital literacy, privacy management, and critical evaluation of online solicitations directly alter the target's profile (Griffith et al., 2023). Applying standard situational crime prevention principles (Felson and Clarke, 1998), school- and parent-led scenario training promotes durable behavioral changes that harden potential targets without criminalizing adolescents. This training reduces target exposure and visibility via strict privacy controls, while fostering skepticism to increase resistance against easy exploitation.

In view of the phenomenon of imitative retaliation and victim-perpetrator transformation among adolescents following online victimization, it is necessary to improve the mechanism of online behavior traceability, impose proportionate penalties, raise the behavioral cost of identity transition, and eliminate their misconception of low-cost violations (Averdijk et al., 2016; Xu, 2025).

### Enhancing multi-party guardianship

Guardianship should be reconceptualized as a distributed, adaptive network that combines technological, institutional, and informal actors—technology providers, families, schools, and law enforcement—so that oversight is continuous across platforms and life-spaces. Technological guardianship entails platform responsibilities such as multi-factor authentication, robust logging, automated monitoring, and adaptive moderation that together raise detection likelihood and shorten response times (DeLiema et al., 2025). Machine-learning tools can materially improve real-time detection of fraudulent or abusive textual patterns, but they demand task-specific tuning, frequent retraining, and adversarial-resilience strategies to remain effective against evolving offender tactics (Papasavva et al., 2025). Informal guardianship—parental mediation, school curricula, and collaborative monitoring tools—is complementary: it sustains day-to-day oversight, builds adolescents' digital resilience, and attenuates the mental-health harms of cyberbullying when parental mediation is high (Hellfeldt et al., 2020; Wright, 2024).

Practical coordination measures—enforcing age limits, synchronizing platform flags with parental or school review workflows, implementing interoperable reporting APIs, and defining clear escalation pathways—preserve guardianship continuity and reduce jurisdictional gaps that offenders exploit. These layered arrangements operate instrumentally by increasing offenders' perceived probability of detection and the expected costs of offending, which empirical evidence suggests may reduce victimization risk (Leukfeldt and Yar, 2016; Sobol et al., 2025). Different types of online victimization require case-specific analysis. In the case of cyberbullying, it is necessary to strengthen platform governance and peer intervention and implement emotional counseling for perpetrators to diminish their sense of impunity. Regarding online fraud, technical measures should fortify payment defenses while expanding family-centric anti-fraud awareness. For privacy violations and online sexual exploitation, it is imperative to strengthen safety education, remediate platform vulnerabilities, and curb personal data leaks among adolescents. Furthermore, addressing the victim–perpetrator overlap, China has deployed effective interventions including real-time, random facial verification and unconditional refunds for minors' unauthorized digital expenditures. This dual-pronged approach systematically raises identity fraud costs, reduces household financial losses, and tightens the joint constraints of guardianship and platform governance.

However, layered guardianship raises governance and ethical challenges—data-sharing agreements, privacy protections, transparent thresholds for automated interventions, management of false positives, and equitable access to protective tools—that require legal mandates, standardized protocols, and accountability mechanisms. To be effective, multi-party guardianship must be adequately resourced, regularly evaluated, and iteratively refined through interdisciplinary collaboration and field testing, so that technical systems, institutional practices, and family-level supports operate as a coherent preventive architecture.

### Disrupting situational convergence

Preventing cyber victimization requires actively breaking the spatiotemporal convergence of a motivated offender, a suitable target, and an absent guardian; one promising strategy is the deployment of big-data early-warning systems that analyze routine-activity traces to detect emerging high-risk interactions before harm crystallizes. By combining temporal clustering, network analysis and anomaly detection, AI models can surface patterns such as sudden increases in soliciting contacts, repeated private messaging from new accounts, or coordinated grooming behaviors and then trigger calibrated responses—automated platform moderation, prioritized human review, targeted alerts to parents, or school outreach—that reduce the co-occurrence of risk factors (DeLiema et al., 2025; Sobol et al., 2025). Meanwhile, big data-based risk early warning should be adopted to screen populations with potential perpetrator tendencies, conduct early intervention and counseling, and prevent the continuous evolution of the vicious cycle between victimization and perpetration. Crucially, these systems must be designed with robust governance: transparent risk thresholds, audited model performance, clear escalation pathways, human-in-the-loop review to curb false positives, and legally enforceable data-sharing agreements to prevent mission creep.

Privacy-preserving technical choices (for example, federated learning, minimization, and differential privacy) and explicit consent, role-based access, and oversight mechanisms are necessary to preserve legitimacy and protect adolescents' rights. From a theoretical standpoint, early intervention serves both RCT and RAT aims: it increases offenders' perceived probability of detection and the expected costs of sanctions while altering routines and exposures that create opportunities for abuse. Empirical evidence from fraud prevention and related domains indicates that disrupting exploitation at its incipient stages may reduce successful attacks, but real-world deployment requires iterative evaluation of predictive validity, equity (differential impacts across age, gender, and socioeconomic groups), and operational burden. In short, data-driven early warning can shrink the high-risk overlap that produces online victimization, provided it is implemented with proportionality, transparency, cross-sector coordination, and continuous monitoring and refinement.

Notably, although risk early warning systems based on AI and big data may help disrupt situational convergence, several challenges related to privacy protection and practical feasibility must be addressed. AI monitoring requires the collection of sensitive information such as behavioral trajectories and social content, which may easily infringe on adolescents' privacy and violate the principle of data minimization (Alghafri and Tubaishat, 2025). Meanwhile, AI models suffer from false alarms, algorithmic bias, adversarial evasion, and other limitations, resulting in delayed identification of emerging harm tactics. Insufficient cross-platform and cross-departmental data collaboration further breaks the continuity of the guardianship chain (DeLiema et al., 2025; Papasavva et al., 2025). Accordingly, early warning systems should adopt technologies such as federated learning and differential privacy, establish a human-in-the-loop review mechanism, clarify intervention thresholds and informed consent procedures, and strike a balance between security assurance and privacy protection (Alghafri and Tubaishat, 2025).

Together, these three dimensions constitute a multi-level, adaptive prevention architecture: raise the costs and barriers to offending while hardening potential targets, sustain distributed guardianship, and intervene early to sever the formation of criminal opportunities. The dual-theory perspective clarifies why these elements must operate in concert: altering offender rationales without changing situational exposure, or vice versa, will leave critical pathways to victimization intact.

For future empirical verification, subsequent quantitative inquiries might consider adopting a multi-stage stratified sampling framework, with research coverage spanning Asia, Europe, Africa, and Latin America. Stratification criteria could include school type, urban-rural residency, age, and gender; future studies could select an appropriately powered sample, for example, approximately 1,200 adolescents, depending on available resources and study design. In terms of statistical analysis, the following parameter settings could be referenced for follow-up research: statistical power 1 – β = 0.80, significance level α = 0.05, and a medium effect size is adopted. Future studies could aim to ensure a sample-to-variable ratio of at least 10:1, while mixed-effects models might serve as a complementary tool to boost statistical power for longitudinal datasets. Before formal analysis, data normality, linearity, independence, and homogeneity of variance should be tested; if assumptions are violated, robust standard errors and data transformation should be applied.

To ensure result reliability, multiple robustness test approaches can be employed in subsequent empirical analyses, including 1,000 bootstrap resamples, outlier exclusion, multi-group comparisons, control for common method bias, and sensitivity analyses using alternative indicators. For the cross-cultural component, scales could undergo bilingual back-translation and cultural calibration. Multi-group confirmatory factor analysis could be performed to test cross-cultural invariance in terms of configural, loading, and intercept invariance, ensuring cross-cultural validity.

Future studies could consider several statistical approaches to examine or test the integrated RAT–RCT theoretical framework; three widely applicable analytical approaches are outlined below for reference only:

- Structural equation modeling: To examine latent variable pathways, mediating effects, and moderating effects of the RAT–RCT integrated framework.
- Longitudinal models: To analyze the temporal causality and developmental trajectories of cyber victimization.
- Hierarchical linear modeling: To account for the individual–environment nested structure and examine cross-level protective and risk factors.

## Conclusion

In this perspective paper, we employ an integrated framework combining RAT and RCT to systematically explore the formation mechanisms of adolescents' online victimization and propose corresponding prevention strategies from multi-stakeholder and multi-level perspectives. Although this research, to some extent, deepens the theoretical understanding of adolescent online victimization and offers practical implications, several limitations remain that future studies should address and build upon.

First and foremost, resting primarily on theoretical deduction and literature synthesis, this study lacks direct empirical verification, particularly regarding the Chinese adolescent population. To establish the generalizability of our model and the effectiveness of the proposed prevention architecture, subsequent research must pursue robust empirical validation. Specifically, future tracking should employ mixed-methods fieldwork, longitudinal cohorts, and experimental designs to clarify how the three RAT elements manifest across varied digital harms and how adolescent cognitive decision-making shapes target suitability. The research scope can be expanded to incorporate more non-Western empirical evidence, while also incorporating longitudinal studies and meta-analytic findings related to online victimization. If this paper is extended into an empirical study using survey methods or statistical modeling, professional statistical support should be introduced to enhance methodological rigor.

Moreover, although the paper emphasizes collaborative governance, it stops short of detailing how responsibilities should be allocated or how cross-sector cooperation should be organized in practice; policy-oriented case studies, comparative policy analysis, implementation pilots, and participatory action research could help construct an institutionalized, operational framework for multi-party collaboration and translate RCT prescriptions—raising costs and reducing rewards—into concrete platform features, regulatory levers, escalation pathways, and incentive structures. Concurrently, rapid technological change (artificial intelligence, the metaverse, deepfakes) continually reshapes offender tactics and creates novel risks that the present discussion does not fully interrogate; systematic horizon-scanning, adversarial threat modeling, and empirical evaluation of technology-enabled prevention (for example, AI moderation, authentication mechanisms, provenance tools, and privacy-preserving early-warning systems) are essential to understand how new tools alter offenders' cost–benefit calculations and affect the practical efficacy of guardianship. In addition, the analysis pays insufficient attention to heterogeneity within the adolescent population: risks and protective factors vary by age, gender, region, socioeconomic status, and family context; stratified sampling, subgroup and intersectional analyses, and fine-grained outcome evaluation will be necessary to segment target populations and to design differentiated, context-sensitive prevention strategies. Taken together, empirical testing, operational governance work, continuous technological appraisal, and nuanced population stratification would strengthen the theoretical claims and make the proposed multi-dimensional prevention system more actionable and equitable.

Based on an integrated perspective of RAT and RCT, this study explores core formative mechanisms of typical adolescent online victimization and constructs a multi-dimensional prevention system. Elevating crime costs, optimizing multi-party guardianship, and cutting off risky situational convergence could mitigate most conventional adolescent online victimization risks, whereas extra targeted supplementary interventions are required for special-type emerging cyber harms. Additionally, future research should focus on several key areas: assessing the impact of emerging technologies on cybercrime and victim prevention; focusing on variations in risk profiles among adolescents of different ages, genders, regions, and family backgrounds (Segura et al., 2020; Sumter et al., 2012), to propose more targeted preventive strategies; enhancing prevention outcomes through interdisciplinary collaboration; exploring the role and mechanisms of international cooperation in preventing cyber victimization; and further exploring victim–perpetrator overlap in adolescent cyber harm, with a focus on gender differences and developmental trajectories among Chinese adolescent samples (Xu, 2025).

## Data Availability

The original contributions presented in the study are included in the article/supplementary material, further inquiries can be directed to the corresponding author.
